# CYFRA21-1 is a more sensitive biomarker to assess the severity of pulmonary alveolar proteinosis

**DOI:** 10.1186/s12890-021-01795-x

**Published:** 2022-01-03

**Authors:** Jiu-Wu Bai, Shui-Yi Gu, Xiao-Li Sun, Hai-Wen Lu, Shuo Liang, Jin-Fu Xu

**Affiliations:** Department of Respiratory and Critical Care Medicine, Shanghai Pulmonary Hospital, Tongji University School of Medicine, No. 507 Zhengmin Road, Shanghai, 200433 China

**Keywords:** Autoimmune pulmonary alveolar proteinosis, Lactate dehydrogenase, Carcinoembryonic antigen, CYFRA21-1, Severity and prognosis score of pulmonary alveolar proteinosis

## Abstract

**Background:**

Serum lactate dehydrogenase (LDH), carcinoembryonic antigen (CEA) and CYFRA21-1 are the commonly used biomarkers to identify patients with autoimmune pulmonary alveolar proteinosis (APAP). However, it is not clear which of the biomarkers is more sensitive to the severity of the patient’s condition.

**Methods:**

APAP patients numbering 151 were enrolled in this study. All patients’ severity was assessed through the severity and prognosis score of PAP (SPSP). According to the respective laboratory upper limits of serum levels of LDH, CEA and CYFRA21-1, APAP patients were divided into higher and lower-level groups. Patients were divided into five groups based on SPSP. 88 patients had completed six months of follow-up. We calculated sensitivity, specificity, and critical point of LDH, CEA and CYFRA21-1 between APAP patients and normal control group, and between grade 1–2 and 3–5 through receiving operating characteristics (ROC) curve.

**Results:**

Serum LDH, CEA and CYFRA21-1 levels of patients with PAP were higher and distinctly related to PaO_2_, FVC, FEV_1_, DLCO, HRCT scores and SPSP. The SPSP of patients in higher-level LDH, CEA and CYFRA21-1 groups were higher than those of corresponding lower-level groups. Based on SPSP results, the patients were divided into five groups (grade I, 20; grade II, 37; grade III, 40; grade IV, 38; grade V, 16). The serum level of CYFRA21-1 of patients with APAP in grade II was higher than that of patients in grade I and lower than that of patients in grade III. Serum CYFRA21-1 of patients with APAP after six months were higher than the baseline among the aggravated group. Serum LDH, CEA and CYFRA21-1 levels after six months among patients in the relieved group of patients with APAP were lower than the baseline. ROC correlating LDH, CEA and CYFRA21-1 values with APAP severity (between grade 1–2 and 3–5) showed an optimal cutoff of LDH of over 203 U/L (< 246 U/L), CEA of over 2.56 ug/L (< 10 ug/L), and CYFRA21-1 of over 5.57 ng/ml (> 3.3 ng/ml) (AUC: 0.815, 95% CI [0.748–0.882], sensitivity: 0.606, specificity: 0.877).

**Conclusion:**

Serum CYFRA21-1 level was more sensitive in revealing the severity of APAP than LDH and CEA levels among mild to moderate forms of disease.

## Background

Pulmonary alveolar proteinosis (PAP) is a rare lung syndrome that was first described in 1958 [1] and characterized by the intra-alveolar accumulation of surfactant lipids and proteins that impair gas exchange, which results in progressive respiratory insufficiency. PAP was divided into three groups [2], namely, congenital PAP, secondary PAP, and autoimmune PAP (APAP). The prevalence of APAP is 0.1 per 100,000 population and accounts for about 90% of all PAP cases [2, 3]. Granulocyte–macrophage colony-stimulating factor (GM-CSF) antibodies were significantly increased in the serum and bronchoalveolar lavage fluid (BALF) of patients with APAP [4] and had a high affinity with GM-CSF and decreased GM-CSF activity [5].

Inoue et al. [6] had suggested the use of the disease severity score (DSS) to assess the severity of PAP and divided the patients five grades on that basis. In 2016, our team proposed the severity and prognosis score of PAP (SPSP) based on smoking status, symptoms, arterial partial pressure of oxygen (PaO_2_), high resolution computed tomography (HRCT) score, and diffusing capacity of the lung for carbon monoxide (DLCO), %predicted [7]. SPSP could more precisely assess the severity and make the prognosis for patients with PAP. The levels of serum lactate dehydrogenase (LDH), carcinoembryonic antigen (CEA) and CYFRA21-1 in patients with APAP were higher than the normal controls [8–11], and the common biomarkers. It was not clear which biomarkers were more sensitive to the severity of APAP among the patients.

## Methods

### Study population

This study was conducted in Shanghai Pulmonary Hospital and consisted of a retrospective cross-sectional analysis from January 2004 to December 2019. During this period, 168 patients were diagnosed with PAP in our institution. Of these, the data of 151 APAP patients were retrospective analyzed in this study after excluding 12 patients with second PAP and five patients whose data was incomplete. Among the enrolled patients, some had comorbidities, such as hypertension and a history of surgery at certain sites, but none of these comorbidities resulted in increased antibodies to GM-CSF. 27 patients were advised to delayed treatment and regular chest CT review, 19 patients refused whole lung lavage (WLL) or GM-CSF therapy, 105 patients received corresponding treatment (WLL: 35; subcutaneous GM-CSF: 49; inhaled GM-CSF: 6; WLL with subcutaneous GM-CSF: 9; WLL with inhaled GM-CSF: 6). Six months’ follow-up data of 88 patients were available. The normal control group was formed with 57 persons who were in the physical examination center. Written informed consent was obtained from all participants through letters. The study protocol was approved by the Ethics Committee of Shanghai Pulmonary Hospital (K19-142; Shanghai, China).

### Diagnostic criteria

Eligibility criteria were selected as described by Ben-Dov et al. [12]. These criteria included histopathologic findings of specimens obtained by open lung biopsy or transbronchial lung biopsy (TBLB); a typical milk-like appearance of BALF, which with lamellar bodies visible in electron microscopy; ground-glass opacity and/or a crazy-paving pattern on HRCT; higher number of GM-CSF antibodies in serum; restrictive ventilation and diffusion dysfunction; clinical symptoms (i.e., dyspnea and cough). The patient would be diagnosed with APAP according to typical HRCT pattern, histopathologic findings or typical BALF, and a higher number of GM-CSF antibodies in serum after excluding other diseases which resulted in GM-CSF antibodies increasing. In this study, the diagnosis of APAP was established by BALF (n = 54), TBLB results (n = 55), or open lung biopsy results (n = 42).

### Interview questionnaire and blood samples

A standardized protocol was used to obtain informed consent from each subject during a medical visit. The interview questionnaire that was used included questions on the following topics: general and anthropometric information (i.e., age and sex), smoking history (e.g., smoker, ex-smoker or never-smoked), and clinical manifestation (e.g., the onset of symptoms, the course of the disease and symptoms). The data about serum LDH, CEA and CYFRA21-1 of patients during the first hospitalization and follow-up in Shanghai Pulmonary Hospital, and normal control group were collected when physical examination. The standard scope of LDH, CEA and CYFRA21-1 were respectively (120–246) U/L, (0–10) ug/L and (0–3.3) ng/ml in our laboratory.

### Severity and prognosis score of PAP (SPSP)

HRCT scans of the chest of 151 patients were analyzed and graded following the visual scoring methods based on two studies [7, 13]. We selected the HCRT grades in four representative regions, namely, the aortic arch, the tracheal carina, the convergence of the left and right inferior lung veins and above the diaphragm. The extent of lung opacity was estimated using a five-point scale: no opacity = 0; opacity involving ≤ 25% of a region of hemithorax = 1; 26–50% = 2; 51–75% = 3; > 75% =  4. The chest HRCT was examined and interpreted independently by two chest physicians. The mean values obtained from the two readers were used for analysis. The chest HRCT score was calculated by summing the lung opacity scores of the four representative regions of each hemithorax.

Pulmonary function was examined in each patient. Forced vital capacity (FVC), forced expiratory volume in 1 s (FEV_1_) and DLCO data were presented as the percentages of predicted values (%predicted). Arterial blood measurements were performed on samples obtained while the patients were breathing room air at rest in the supine position.

The severity of patients was assessed based on the SPSP [6]. SPSP included smoking statues (never smoker, 0; smoker, 1); symptoms (No, 0; Yes, 1); PaO_2_ (≥ 80 mmHg, 0; ≥ 60 mmHg and < 80 mmHg, 1; < 60 mmHg, 2); HRCT score (≤ 8, 1; > 8 and ≤ 16, 2; > 16 and ≤ 24, 3; > 24, 4); and DLCO, %predicted (≥ 80%, 0; ≥ 60% and < 80%, 1; < 60%, 2). The SPSP was from 1 to 10. According to the SPSP, the patients were divided into five grades (grade I, SPSP 1–2; grade II, SPSP 3–4; grade III, SPSP 5–6; grade IV, SPSP 7–8; grade V, SPSP 9–10), and three severity grades: mild (grade I and II), moderate (grade III) and severe (grade IV and V).

### Statistics

SPSS version 19.0 (SPSS, Chicago, Illinois) was used for statistical analysis. The data were tabulated as the means and standard deviations for quantitative variables or as absolute numbers and percentages for qualitative variables. The Kolmogorov–Smirnov test was used to analyze the data distribution for each variable. Serum biomarkers (LDH, CEA and CYFRA21-1) were comparatively analyzed between APAP patients and the normal control group. The relations between serum biomarkers and SPSP were analyzed. APAP patients were divided into higher and lower-level groups based on the comparison with the laboratory upper limit of serum biomarkers. The SPSP of patients in the corresponding two groups was comparatively analyzed. Serum biomarkers were pairwise comparatively analyzed among grade 1–5 groups. The 88 patients who had been followed up for six months were divided into three groups (aggravated group, relieved group and stable group) based on SPSP results after 6 months of follow-up as compared to the baseline. The variations in the serum biomarkers between the baseline and after 6 months in each group were subjected to comparative analysis. For the variables, we calculated sensitivity, specificity and critical point of biomarkers between APAP patients and normal control group, and between grades 1–2 and 3–5. We plotted the receiving operating characteristics (ROC) curve. In the bivariate analysis, the student’s t-test for independent variables was used to analyze variables that were normally distributed, and the Mann–Whitney U test was used to analyze variables that were non-normally distributed. Qualitative variables were compared using the chi-square test. *P* < 0.05 was considered indicative of a significant difference.

## Results

**Fig. 1:**
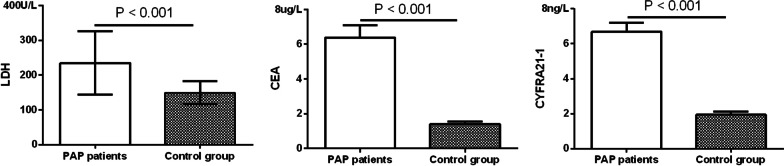
Serum biomarkers between APAP patients and normal control group. Serum LDH, CEA and CYFRA21-1 of patients with PAP were higher than normal control group (all *P* < 0.01)

**Fig. 2:**
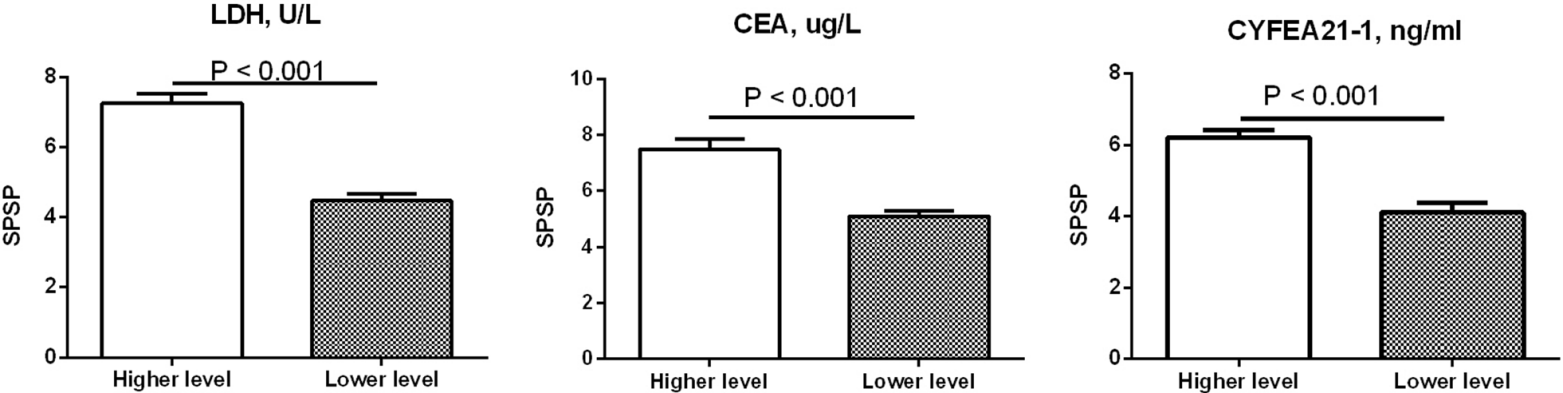
The SPSP of patients in higher and lower level groups on serum biomarkers. The SPSP of patients in higher serum LDH, CEA or CYFRA21-1 groups was higher than that of corresponding lower level groups (all *P* < 0.001)

There was no apparent difference in age and sex ratio between APAP patients and the normal control group. The mean onset age of patients was 45.8 years, male patients accounted for 71.5%, the main symptoms included cough and dyspnea (Table 1). Serum levels of LDH, CEA and CYFRA21-1 among patients with APAP were higher than of the normal control group (all *P* < 0.01) (Fig. 1), and distinctly related to PaO_2_, FVC, FEV_1_, DLCO, HRCT score and SPSP (Table 2). Many patients had abnormally high levels of LDH (> 246 U/L), CEA (> 10 ug/L) and CYFRA21-1(> 3.3 ng/ml) in serum (53, 35.1%; 23, 15.2%; 97, 64.2%). The SPSP was higher among patients in the group with higher-serum levels of LDH, CEA or CYFRA21-1 than that of patients in the corresponding lower-level groups (all *P* < 0.001) (Fig. 2).

**Table 1:** The characteristics of patients with PAP and control group

Group	PAP group	%	Control group	%	*P* value
Subjects, n	151		57		
Age, years	47.3 ± 11.6		47.9 ± 13.4		0.731
Sex					
Male	108	71.5	40	70.2	0.849
Female	43	28.5	17	29.8	
Onset age, years	45.8 ± 11.5				
Course of disease, months	18.2 ± 25.6				
Smoking status	64	42.4			
Symptoms					
Asymptomatic	16	10.6			
Cough	109	72.2			
Dyspnea	91	60.3			
Chest pain	9	6.0

**Table 2:** The relation coefficient between serum biomarkers and PAP severity

	LDH, U/L	CEA, ug/L	CYFRA21-1, ng/ml
PaO_2_	− 0.346	− 0.324	− 0.368
FVC	− 0.341	− 0.270^a^	− 0.285
FEV_1_	− 0.384	− 0.241^b^	− 0.327
DLCO	− 0.397	− 0.290	− 0.435
HRCT score	0.427	0.393	0.516
SPSP	0.440	0.384	0.513

^a^The relation coefficient between CEA and FVC (*P* = 0.001). ^b^The relation coefficient between CEA and FVC (*P* = 0.003); other all *P* < 0.001

**Fig. 3:**
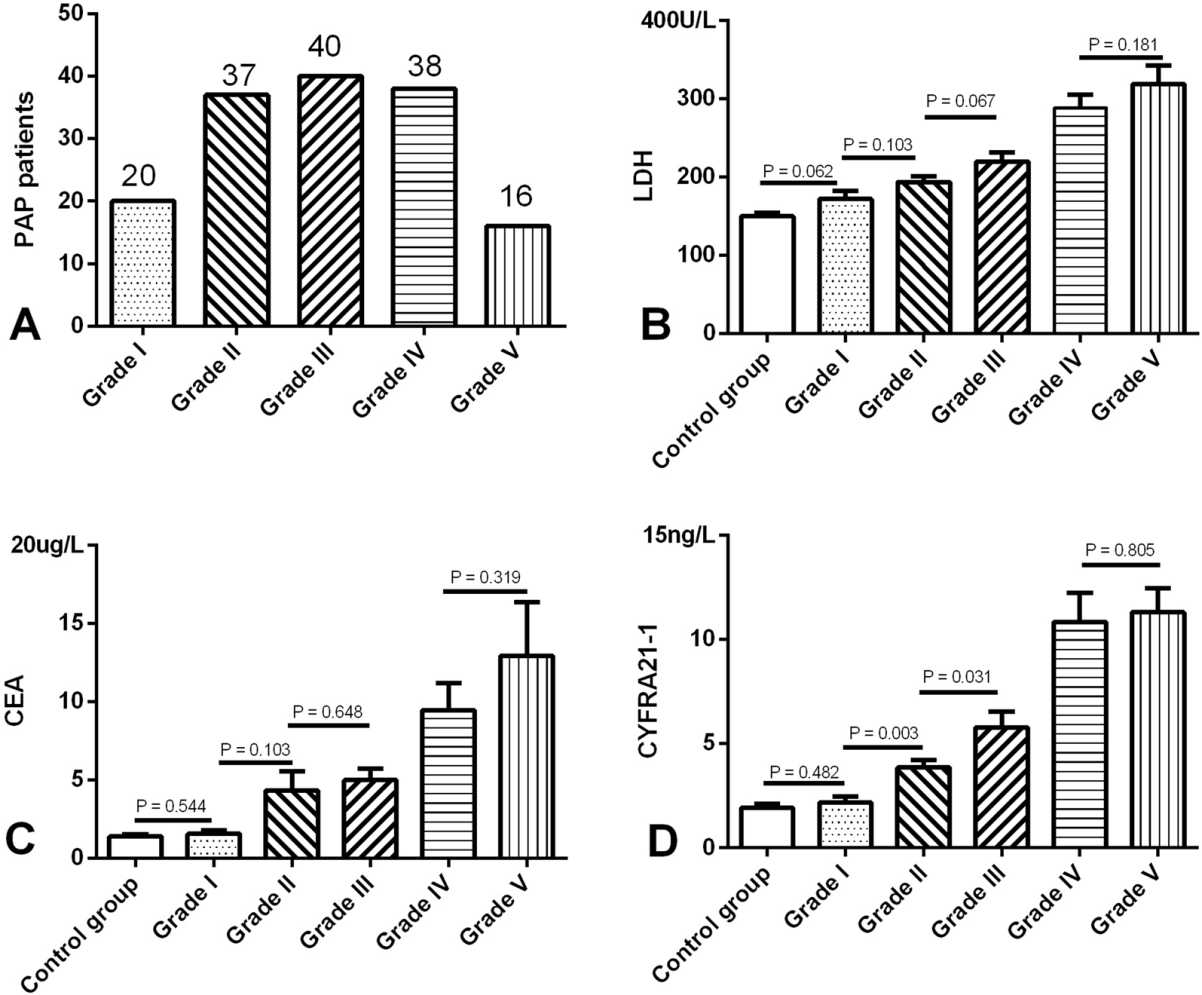
Serum biomarkers during different grade PAP patients. **A** Grade I, 20; grade II, 37; grade III, 40; grade IV, 38; grade V, 16. *P* value: all *P* > 0.05(serum LDH, CEA and CYFRA21-1 between Grand I and control group); all *P* > 0.05(serum LDH and CEA between Grand II and I or III), *P* = 0.003 (serum CYFRA21-1 between Grand I and II), and *P* = 0.031(serum CYFRA21-1 between Grand II and III); all *P* > 0.05(serum LDH, CEA and CYFRA21-1 between Grand IV and V); All the other *P* < 0.05 (serum LDH, CEA and CYFRA21-1 between other any two groups)

Based on SPSP results, the patients were divided into five groups (grade I, 20; grade II, 37; grade III, 40; grade IV, 38; grade V, 16) (Fig. 3A). Serum level of CYFRA21-1 of patients with APAP in grade II was higher than grade I (*P* = 0.003) and lower than grade III (*P* = 0.031), but there was no apparent difference in serum levels of LDH and CEA between grade II and grades I or III (all *P* > 0.05). There was a marked difference in serum LDH, CEA and CYFRA21-1 levels between any other two groups (all *P* < 0.05) (Fig. 3) except between grade I and control group and between grades IV and V.

**Fig. 4:**
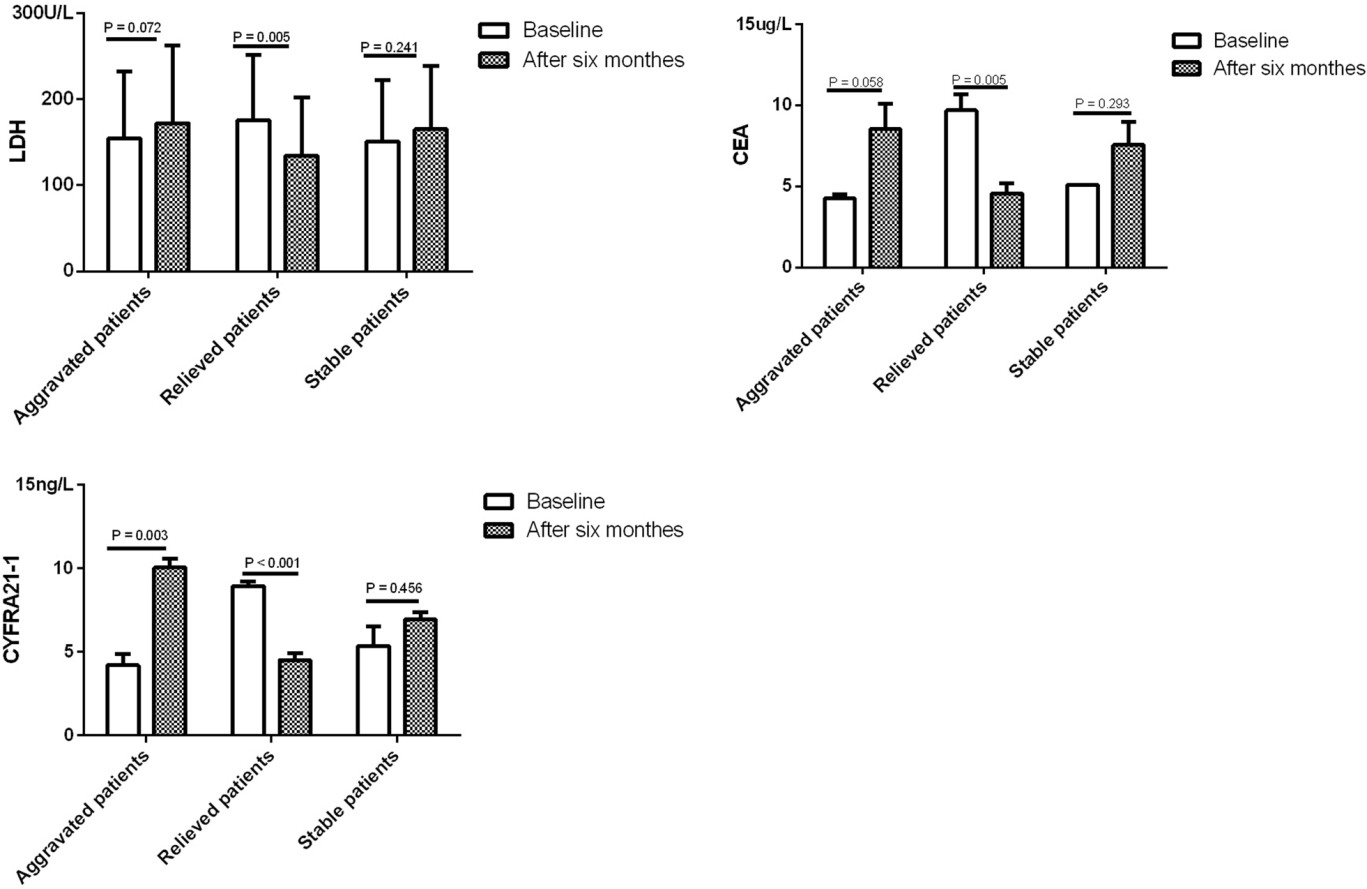
Serum biomarkers in APAP patients between before treatment and after six months. Serum CYFRA21-1 of patients with PAP after six months were higher than baseline in aggravated group (*P* = 0.003). Serum LDH, CEA and CYFRA21-1 of patients with PAP after six months were lower than baseline in relieved group (*P* = 0.005, 0.005; *P* < 0.001)

The 88 followed-up patients had been divided into the aggravated group (n = 27), relieved group (n = 40) and stable group (n = 21). In the aggravated group, the serum levels of CYFRA21-1 of patients with APAP after six months were higher than the baseline (*P* = 0.003). Serum LDH, CEA and CYFRA21-1 levels of patients with APAP after six months were lower than baseline in relieved group (*P* = 0.005, 0.005; *P* < 0.001) (Fig. 4).

**Fig. 5:**
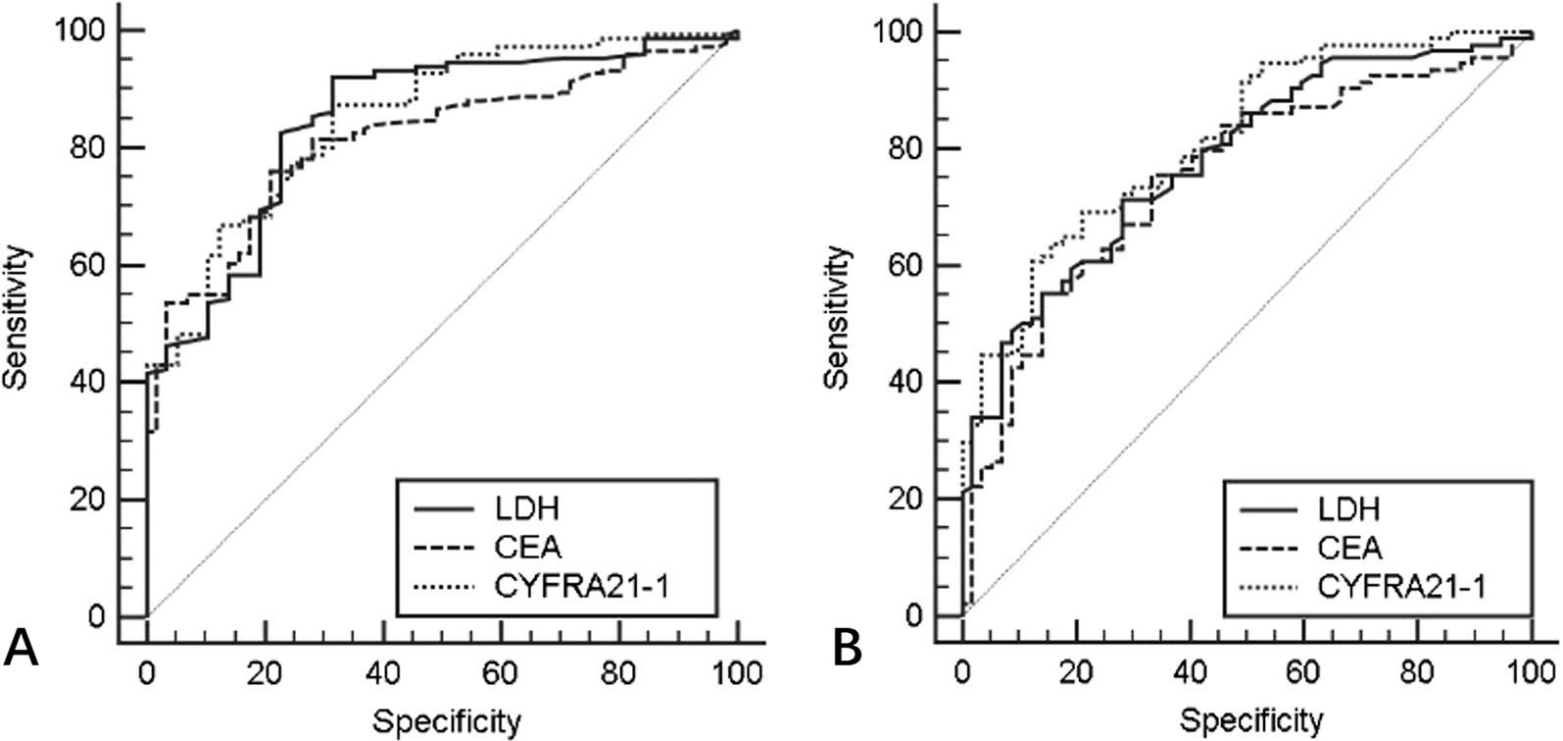
Receiver operating characteristic curve (ROC) of correlating serum biomarkers. **A** LDH value and PAP (critical point: 146 U/L, AUC: 0.848, 95% CI [0.791–0.906], sensitivity: 0.921, specificity: 0.684); CEA value and PAP (critical point: 1.47 ug/L, AUC: 0.815, 95% CI [0.757–0.874], sensitivity: 0.762, specificity: 0.789); CYFRA21-1 value and PAP (critical point: 2.01 ng/ml, AUC: 0.852, 95% CI [0.797–0.906], sensitivity: 0.874, specificity: 0.684).** B**: LDH value and PAP severity (critical point: 203 U/L, AUC: 0.780, 95% CI [0.707–0.853], sensitivity: 0.713, specificity: 0.719); CEA value and PAP severity (critical point: 2.56 ug/L, AUC: 0.750, 95% CI [0.671–0.829], sensitivity: 0.755, specificity: 0.659); CYFRA21-1 value and PAP severity (critical point: 5.57 ng/ml, AUC: 0.815, 95% CI [0.748–0.882], sensitivity: 0.606, specificity: 0.877)

ROC correlating serum biomarkers’ (LDH, CEA and CYFRA21-1) values with APAP was plotted as shown in Fig. 5A. It shows an optimal cutoff of LDH at over 146 U/L (AUC: 0.848, 95% CI [0.791–0.906], sensitivity: 0.921, specificity: 0.684), CEA at over 1.47 ug/L (AUC: 0.815, 95% CI [0.757–0.874], sensitivity: 0.762, specificity: 0.789), and CYFRA21-1 at over 2.01 ng/ml (AUC: 0.852, 95% CI [0.797–0.906], sensitivity: 0.874, specificity: 0.684). ROC correlating LDH, CEA and CYFRA21-1 value and APAP severity (between grades 1–2 and 3–5) was also plotted as presented in Fig. 5B, showing an optimal cutoff of LDH at over 203 U/L (AUC: 0.780, 95% CI [0.707–0.853], sensitivity: 0.713, specificity: 0.719), the cutoff of CEA at over 2.56 ug/L (AUC: 0.750, 95% CI [0.671–0.829], sensitivity: 0.755, specificity: 0.659), and the cut-off of CYFRA21-1 of over 5.57 ng/ml (AUC: 0.815, 95% CI [0.748–0.882], sensitivity: 0.606, specificity: 0.877). Based on serum CYFRA21-1 level, APAP patients were divided into three groups: i ≤ 3.3 ng/ml (54/151); ii < 3.3 ng/ml and ≤ 5.6 ng/ml (33/151); iii > 5.6 ng/ml (64/151). SPSP in iii group (7.0 ± 1.9) was far higher than in group i (4.1 ± 1.9) and group ii (4.6 ± 2.0) (both *P* < 0.001), but there was no difference between groups i and ii (*P* = 0.251).

## Discussion

In previous papers, LDH, CEA, CYFRA21-1 and KL-6 were studied to assess their relationship with the severity of patients with APAP [8, 9, 14, 15]. However, serum KL-6 was not a routine inspection in China. As an early assessment criterion, DSS was only a grading criterion and has not for obtaining specific scores. SPSP was more detailed and had definite scores and could be used to assess the relationship between those markers and the severity of patients’ APAP. Some studies noted that a small proportion of APAP patients experienced spontaneous remission [7, 10], not all APAP patients needed a particular treatment (whole lung lavage or inhalation GM-CSF). If the SPSP was less than 5, the patient was not advised some particular treatment [7].

In previous studies [11, 16], LDH, CEA and CYFRA21-1 may have been considered as important indices for monitoring the severity of patients with APAP. In this study, serum LDH, CEA and CYFRA21-1 levels of patients with APAP were higher than the normal control group and distinctly related to SPSP. This result indicated LDH, CEA and CYFRA21-1 may be regard as biomarkers to assess the severity of patients with APAP. In 1991, serum LDH had been noted to be elevated in patients with APAP, and it was decreased through lavage [16]. CEA and CYFRA21-1 were regarded as disease severity markers for APAP in some studies [9, 15].

Based on the upper limit of serum biomarkers in our clinical laboratory, we found that patients with elevated LDH were only 35.1%; the percentage of patients with higher-level CEA was only 15.2%; the SPSP score of these patients placed them in grade IV and V based on the combination of Figs. 2 and 3. In previous studies, the abnormally high level of LDH in serum was 51–62% [9, 17], that of serum CEA was 49–63% [17, 18]. The percentage of elevated LDH and CEA in patients with APAP in different studies were different, this result may be related to the number and the severity of enrolled patients and different laboratory standard. In this study, elevated CYFRA21-1 patients accounted for 64.2%. This percentage was not reported in the past.

Patients with APAP were divided into five grades per 2 scores based on the SPSP. In the previous study, our team suggested the patient whose SPSP was less than 5 would undergo follow-up assessments. Among the grades I, II and III groups, the levels of serum LDH or CEA were not markedly different between two adjacent groups, but significantly different between two nonadjacent groups. There was a significant difference in serum CYFRA21-1 between grade II and grade I or grade III. The above results indicated serum CYFRA21-1 was more sensitive and suitable than LDH and CEA for distinguishing the severity of mild to moderate APAP patients. In patients with severe APAP, there were no differences among the levels of LDH, CEA and CYFRA21-1 between patients in grades IV and V patients.

Of the three groups into which the 88 followed-up APAP patients were divided based on SPSP, in the relieved group, serum LDH, CEA and CYFRA21-1 levels in patients after six months of follow-up were lower than the baseline. For the aggravated group, serum CYFRA21-1 levels in patients with APAP after six months were higher than the baseline, but the levels of LDH and CEA were not higher. These results showed that CYFRA21-1 was preferable for assessing the severity of APAP among patients. This finding is like findings from other studies [15].

According to the results from ROC analysis, the cutoff of serum levels of LDH, CEA and CYFRA21-1 to distinguish patients with APAP and normal people were respectively 146 U/L, 1.47 ug/L and 2.01 ng/ml. Those results were much lower than the upper normal values (246 U/L, 10 ug/L and 3.3 ng/ml). These results caused the values of serum LDH, CEA and CYFRA21-1 to be easily neglected as the means of distinguishing patients with APAP from normal people. In the report by Arai et al., the cut-off level of serum CYFRA 21-1 to diagnose APAP was 3.80 ng/mL, which was higher than 3.3 ng/ml, but the study only enrolled 48 patients. The difference may be related to the number and severity of APAP in the enrolled patients. The cutoff of serum LDH and CEA to distinguish different severity (grade I to II vs. grade III to V) were respectively 203 U/L and 2.56 ug/L which were lower than the upper normal value; while the cut-off of serum CYFRA21-1 was 5.57 ng/ml that was higher than 3.3 ng/ml. SPSP of patients with APAP (serum CYFRA21-1 > 5.6 ng/ml) was far higher than the other two groups (≤ 3.3 ng/ml and < 3.3 ng/ml and ≤ 5.6 ng/ml). This phenomenon further indicated that CYFRA21-1 may serve as an extremely important indicator for identifying the patients who need a particular treatment. The upper limits of LDH, CEA and CYFRA21-1 in the clinical laboratory would be adjusted to patients with APAP.

Surfactant synthesis and secretion were restricted to alveolar epithelial cell type II, and their clearance depended on II cells and macrophages [19]. GM-CSF could regulate surfactant catabolism in alveolar macrophages [20] and suppress the apoptosis of alveolar epithelial cells [21]. Higher levels of anti-GM-CSF autoantibodies cause deficiency of GM-CSF, which leads to the dysfunction of the alveolar epithelial barrier and increasing their permeability. CYFRA21-1 is expressed in hyperplastic type II pneumocytes. This type of hyperplasia was observed in the samples from the lungs of APAP patients [15]. This may be the reason why the production of CYFRA 21-1 increased, but the pathophysiology was unknown.

The main limitation of our study was that this study was a retrospective study. The mechanism that caused the increase in these biomarkers is unknown and needs more research at the signal transduction level.

## Conclusions

Serum levels of LDH, CEA and CYFRA21-1 in the patients with APAP were related to the severity of APAP. Serum CYFRA21-1 level was more sensitive to the severity of APAP than LDH and CEA levels among mild to moderate patients. The cutoff of serum levels of LDH, CEA and CYFRA21-1 to distinguish patients with APAP from normal people were much lower than the upper normal value. The cutoff of serum CYFRA21-1 was 5.57 ng/ml to distinguish different degrees of severity of APAP among patients.

## Data Availability

The datasets used and/or analyzed during the current study are available from the corresponding author on reasonable request.

## Abbreviations

APAP: Autoimmune pulmonary alveolar proteinosis
BALF: Bronchoalveolar lavage fluid
CEA: Carcinoembryonic antigen
DLCO: Diffusing capacity of the lung for carbon monoxide
FEV_1_: Forced expiratory volume in 1 s
FVC: Forced vital capacity
GM-CSF: Granulocyte–macrophage colony-stimulating factor
HRCT: High resolution computed tomography
LDH: Lactate dehydrogenase
PaO_2_: Arterial partial pressure of oxygen
PAP: Pulmonary alveolar proteinosis
ROC: Receiving operating characteristics
SPSP: Severity and prognosis score of PAP
TBLB: Transbronchial lung biopsy
